# Whole-Genome Sequence of *Myxococcus* Phage Mx9

**DOI:** 10.1128/mra.00221-23

**Published:** 2023-05-23

**Authors:** Sébastien Wielgoss, Bryan Julien

**Affiliations:** a Institute of Integrative Biology, Department of Environmental Systems Science, ETH Zürich, Zürich, Switzerland; b Private address, Zionsville, Indiana, USA; Portland State University

## Abstract

Here, we characterize the genome of *Myxococcus* phage Mx9, a lysogenic, short-tailed phage (genus *Lederbergvirus*) phage infecting the bacterial host Myxococcus xanthus, a model for bacterial evolution and development. The 53.5-kb genome has a GC content of 67.5% and contains 98 predicted protein-coding genes, including the previously characterized site-specific integrase gene (*int*).

## ANNOUNCEMENT

The myxobacterium Myxococcus xanthus is a soilborne microbial predator (1) and serves as a model for evolution and development (2). Many functions of its multicellular life cycle have been elucidated in molecular detail (3–15). Several bacteriophages of M. xanthus were isolated (16), of which two were fully sequenced, namely, *Myxococcus* phages Mx4 (17) (GenBank accession number OK085710) and Mx8 (GenBank accession number AF396866). Here, we report the whole-genome sequence of the general transducing *Myxococcus* phage Mx9. The short-tailed Mx9 particle is serologically distinct from but morphologically similar to Mx8 (18). Mx9 integrates its double-stranded DNA into the host genome via the phage-encoded integrase gene, at either of two *attB* sites (19). A high-titer stock of Mx9 was prepared as described previously (19). In brief, Mx9 was reisolated from its lysogen (DK816) by plating dilutions of the bacterial culture supernatant onto the susceptible host strain M. xanthus DZ1. A single Mx9 plaque was mixed with ~5 × 10^8^ CFU/mL of the susceptible host strain M. xanthus DZ1 in 0.5 mL CTS broth (1% Casitone, 0.2% MgSO_4_ · 7H_2_O, 50 mM HEPES; pH 7.6). This mixture was incubated for 20 min at room temperature without shaking and then embedded in 2.5 mL soft agar; after 2 days of incubation at 30°C in 90% relative humidity, the lysed lawn was overlaid with 5 mL of phage buffer (19) for 24 h at 4°C for harvesting of free phages.

Genomic DNA was extracted via proteinase K (20 mg/mL) treatment followed by phenol-chloroform-isoamyl alcohol extraction steps (19). The DNA was resuspended in buffer EB (Qiagen) and mechanically sheared to ~350-bp fragments using a multifunctional bioprocessor (EpiSonic), from which a sequencing library was prepared with the NEBNext Ultra DNA kit for sequencing on a NovaSeq 6000 instrument (in 151-bp paired-end mode). Sequencing reads were quality checked with FastQC v0.11.8 (20) and trimmed (minimum length, 36 bp) using Trimmomatic v0.32 (21) (see Table 1 for read statistics). Trimmed reads were used for assembly in SPAdes v3.11.1 (22) (parameters: –k 21,33,55,77,99,127 –careful). The assembly resulted in 3,055 contigs, of which all but one were removed due to short lengths (length of <1,000 bp) and/or low k-mer coverage (coverage of <3). The retained sequence (~54 kb, with k-mer coverage of 4,008) was subsequently polished with Pilon (23) using the corrected read set from the SPAdes assembly. Importantly, the contig features the complete, previously sequenced (19) Mx9 integrase region (GenBank accession number AY247757) (~4.6 kb) with a perfect match.

**TABLE 1:** Statistics for Illumina sequencing reads

Parameter	Finding
SRA library name	Mx9
SRA accession no.	SRR23870516
No. of raw reads	14,680,546
No. of trimmed reads	12,661,869
Avg raw read length (bp)	151
Avg trimmed read length (bp)	134.5
Size of raw sequence (Gbp)	2.22
Size of trimmed sequence (Gbp)	1.70

**FIG 1:**
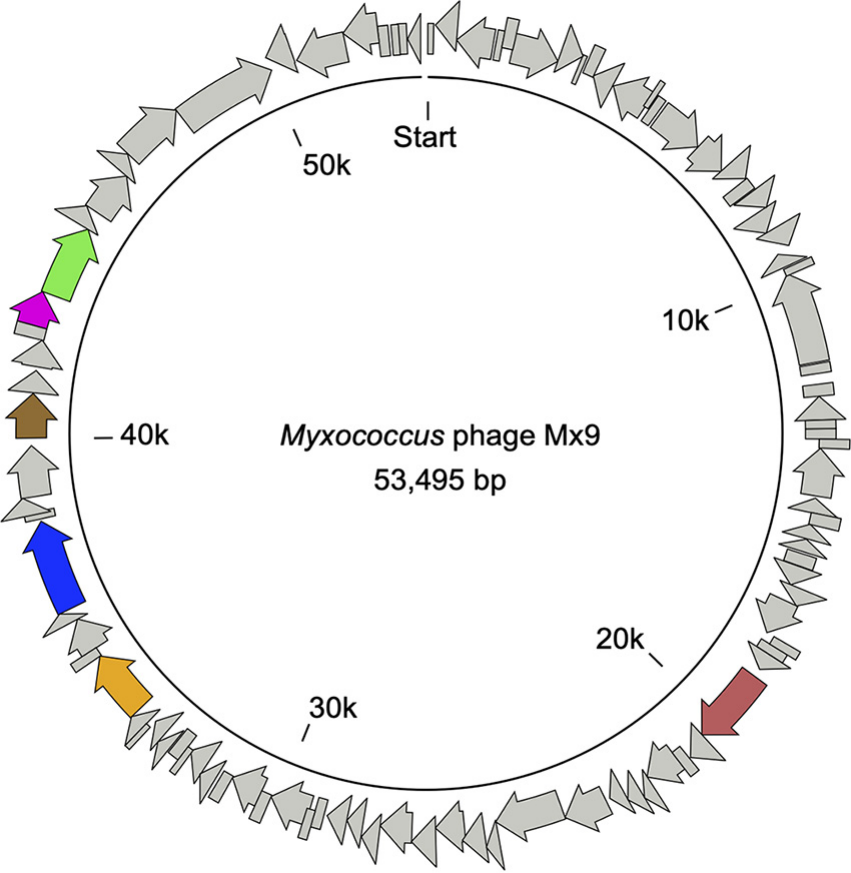
Open circle representation of the linear *Myxococcus* phage Mx9 genome. The genome contains 98 protein-coding genes (arrows). Coloration is based on protein classification using VIRFAM (27), as follows: maroon, site-specific integrase Int (Mx9_p42); orange, phage terminase TermL, large subunit (Mx9_p73); blue, portal protein (Mx9_p77); brown, major capsid protein (Mx9_p81); magenta, adaptor protein (Mx9_p85); green, head-closure protein (Mx9_p86). Inner ring labels show nucleotide positions in kilobase pairs.

The finalized assembly is 53,495 bp long, with 63,283-fold nucleotide coverage and a GC content of ~67.5% (Fig. 1). PhageTerm (24) analysis predicted that the genome is linear and has a single obvious terminus on the forward strand (headful DNA packaging [PAC]), with terminal redundancy. We performed gene annotation with the bacteriophage option in BV-BRC (25) to predict protein-coding genes and taxonomy (identified as *Lederbergvirus*). After manually curating the boundaries of the previously described integrase gene (*int*), we found a total of 98 predicted protein-coding sequences, with a coding density of ~96.5%. We performed profile-to-sequence comparisons of our predicted proteins against the protein orthologous groups (PHROG) database to improve gene annotations (26). According to head-neck-tail protein classification in VIRFAM (27), Mx9 forms a sister group with *Myxococcus* phage Mx8, which is in line with previous predictions (18, 19).

### Data availability.

Genome sequencing was performed at Oxford Genomics (Oxford, UK). The genome sequence was deposited in DDBJ/ENA/GenBank under accession number OQ709411. Raw sequencing reads were deposited in the Sequence Read Archive (SRA) under accession number SRR23870516.
